# An overview to the investigative approach to species testing in wildlife forensic science

**DOI:** 10.1186/2041-2223-2-2

**Published:** 2011-01-13

**Authors:** Adrian Linacre, Shanan S Tobe

**Affiliations:** 1 South Australia Justice Chair in Forensic Science, School of Biological Sciences, Flinders University, Adelaide, South Australia, Australia; 2 Centre for Forensic Science, WestChem, University of Strathclyde, Glasgow, UK

## Abstract

The extent of wildlife crime is unknown but it is on the increase and has observable effects with the dramatic decline in many species of flora and fauna. The growing awareness of this area of criminal activity is reflected in the increase in research papers on animal DNA testing, either for the identification of species or for the genetic linkage of a sample to a particular organism. This review focuses on the use of species testing in wildlife crime investigations. Species identification relies primarily on genetic loci within the mitochondrial genome; focusing on the cytochrome *b* and cytochrome oxidase 1 genes. The use of cytochrome *b* gained early prominence in species identification through its use in taxonomic and phylogenetic studies, while the gene sequence for cytochrome oxidase was adopted by the Barcode for Life research group. This review compares how these two loci are used in species identification with respect to wildlife crime investigations. As more forensic science laboratories undertake work in the wildlife area, it is important that the quality of work is of the highest standard and that the conclusions reached are based on scientific principles. A key issue in reporting on the identification of a particular species is a knowledge of both the intraspecies variation and the possible overlap of sequence variation from one species to that of a closely related species. Recent data showing this degree of genetic separation in mammalian species will allow greater confidence when preparing a report on an alleged event where the identification of the species is of prime importance. The aim of this review is to illustrate aspects of species testing in wildlife forensic science and to explain how a knowledge of genetic variation at the genus and species level can aid in the reporting of results.

## Scope of wildlife crime

Wildlife crime takes many forms from trafficking in live specimens, hunting out of season, cruelty to animals, habitat destruction, poaching for meat, poaching for trophies, poaching to use animal parts in medicines, horns and tusks used for jewellery and ornaments - the list goes on. The scope of wildlife crime covers a wide range of diverse crimes and for this reason many newspaper articles, as well as journal papers, will often cite figures such as:

'The illegal trade in wildlife is a $20 billion a year industry, second only to trade in illegal drugs'.

The monetary figure will often range between 6 and 20 billion US dollars a year and the figure is often cited to Interpol [1]. However, Interpol have confirmed that this statement did not come from them. While this seems to be a fabricated figure, it is difficult to estimate the exact amount of illegal trade as there are not the same international surveillance teams that are used for drug enforcement for the prosecution of offences involving wildlife. Organized crime has not been proven to be linked to wildlife crime but there are indications that this is the case [2]. Another influencing factor in wildlife crime is that there is a high financial return with little chance of being caught and, even if the perpetrators are caught, the penalties are light. Rarely does the maximum penalty for the alleged event meet the potential financial gains [3].

According to a recent census by the World Wildlife Fund only 3200 tigers (*Panthera tigris *spp.) exist in the wild [4]. This is a reduction of over 90% in the last century which has lead to more tigers existing in captivity in Texas than exist worldwide in the wild. Similarly, the population of black rhino (*Diceros bicornis*) decreased by 96% between 1970 and 1992 [5]. In 1970, it was estimated that there were approximately 65,000 black rhinos in Africa - but, by 1993, there were only 2300 surviving in the wild. Intensive anti-poaching efforts have had encouraging results since 1996. The numbers of black rhino have been recovering and still are increasing very slowly; there are now an estimated wild population of 4420.

The above examples illustrate the affect of trade on the numbers for the tiger and rhino populations. The biological material that is traded is not the whole animal but body parts such as skin, bone or powdered horn. Other examples of mammalian species that are part of the illegal trade in wildlife include elephant ivory [6-10], bear bile [11] and deer products [12-14]. Mammalian species are high profile in the public perception but the trade in reptiles and amphibians is much higher, partly because these species are smaller and therefore easier to conceal in order to avoid detection [15].

A paradox to the limited prosecutions is the rise in interest in the forensic community in wildlife forensic science. There have been reviews of the subject [16-19], a text book on non-human DNA [20] and on wildlife forensic science [21]. There are more publications on non-human DNA in the international journal *Forensic Science International: Genetics *than papers on topics such as single nucleotide polymorphisms, mixtures or low template DNA typing [22]. Given this interest, it is noteworthy that there are very few laboratories dedicated to wildlife forensic science; the major exception being the US Fish and Wildlife Laboratory http://www.lab.fws.gov in Oregon, USA.

## Forensic science relating to wildlife crime

There are two prime issues that are addressed in wildlife crime and these relate to the phrasing of the different types of legislation. The first being the ability to identify a particular species and the second is the ability to determine whether the biological material can be assigned with confidence to a particular individual member of that species. This review focuses on this first issue.

The reason for determining a particular species is that many species are listed as being protected both at a national and international level. There are currently 175 countries that are signatories to the Convention for the International Trade in Endangered Species of Flora and Fauna (CITES) - an organization that oversees the movement of protected and endangered species across international borders [23,24]. Each member country is responsible for the implementation of the Convention at a national level. National legislation has been enacted in many countries which is specifically aimed at the protection of species within their own country. Examples include the Endangered Species Act 1973 in the USA [25], which covers alleged crimes at a federal level. Such all encompassing legislation may include additions and amendments, such as the Wildlife and Countryside Act 1981 in the UK [26] with amendments in 1985 [27] and 1991 [28], with further legislation covering deer [29], seal [30] and badger [31]. Prosecutors are tasked with investigating any alleged transgression of the legislation and, hence, require a scientific test in order to identify a particular species. The test employed depends on the material seized and the funds available for conducting the test. As much of the trade in endangered species originates in countries where funds to enforce any crime are limited, the prosecution of wildlife crime can be given a low priority.

Morphology and microscopy are the natural starting points in identification [32]. This depends on the sample seized but there is little point in requesting molecular testing if the material seized is clearly that of a tiger skin, a section of elephant ivory or a specific shell of a tortoise. Morphological analysis of parts of an animal, or even a live specimen, will often have to be undertaken by a specialist, often from a zoo or a museum. Microscopy of hairs is another skill requiring much experience in order to be able to identify with a high degree of confidence that the material is, for instance, that of a protected species such as the Tibetan antelope (*Pantholops hodgsonii*), which is protected and CITES listed, compared to those species that are not protected. However, even with experience, a microscopic comparison of hairs may not yield a definitive identification. In Moore's key for the identification of animal hairs, dog appears in over 10 categories and most identifications finish in a group of organisms (for example, camel or dog or llama group) [33]. Much material that is traded is not in a condition where species identification can be made by microscopy or morphology - for instance, the material may present as powders, potions and oils. Biochemical and molecular methods using antibodies or DNA are the tools that can assist with such investigations.

## Role of DNA

The application of DNA-based technologies to the investigation of wildlife crime has opened up the possibility of examining trace material [32]. For instance, in a case where microscopy was used for the putative identification of the Tibetan antelope from woven shawls, the identification to species level can be conducted using DNA typing [34] where the results are not based on the subjective judgement of the examiner. One problem is the associated cost of DNA profiling compared to microscopy. The DNA-based methods used in wildlife crime investigations were adapted from those used in human identification and, in the case of species identification, from taxonomic and phylogenetic studies. For several reasons, the DNA loci used in species testing are located on the mitochondrial genome rather than being nuclear DNA based. Mitochondrial DNA typing has become a standard process in species testing, allowing inter-laboratory comparison and permitting a means to standardize methodologies.

The mitochondrial genome in eukaryotes encodes a total of 37 genes, 22 of which encode transfer RNA (tRNA) molecules, two encode ribosomal RNA (rRNA) molecules and the other 13 encode proteins involved primarily with the process of oxidative respiration [35]. The number of genes on the mitochondrial genome is largely invariant for all vertebrate mitochondrial genomes but the order of the genes may alter [36]. The order of the loci on the mitochondrial genome is the same within mammalian species but can differ between taxonomic classes: for instance the order is different between avian and mammalian mitochondrial genomes. Vertebrate mitochondrial DNA has two strands of different buoyant densities: the heavy or H-strand and the light or L-strand. The H-strand is the sense strand for one protein-coding gene (ND6) and eight tRNA genes. The L-strand is the sense strand for 12 protein-coding genes, two rRNA genes and 14 tRNA genes [37].

A major reason for using mitochondrial DNA (mtDNA) loci is that there is no recombination of mtDNA. All maternal descendents will have the same mitochondrial DNA sequence, with the exception of mutations, and all loci will be linked [38,39]. There is little DNA on the mtDNA that is non-coding, nor are there introns or pseudogenes within the mammalian mtDNA [36]. With all the coding sections of the mitochondrial genome coding for proteins or RNA molecules involved in respiration, it would be expected that there would be conservation of sequence as any change in the proteins or RNA molecules could adversely affect the organism. Unlike the nucleus, no error reading enzyme exists in the mitochondria to correct DNA bases added incorrectly during DNA replication [40]. Therefore, the accumulation of single base changes is up to five times higher in mtDNA compared to errors due to DNA replication in nuclear DNA.

Additionally, there are multiple copies of mitochondrial DNA per cell compared to only two copies of nuclear DNA [41]. Within each cell there are multiple mitochondria depending on cell type and within each mitochondrion there are multiple copies of mtDNA [42]. The result is that there can be many thousands of mtDNA copies in each cell [42]. Mitochondria have a protein coat that helps protect the mtDNA from degradation. Highly degraded biological material is therefore more likely to be amenable to mtDNA typing compared to the need to generate a DNA profile from nuclear DNA when typing teeth [43,44], bone [45,46] or hair shafts [47,48]. Ancient DNA studies have centred on mtDNA for this purpose with such techniques being familiar to the forensic science community for identification of human remains [49,50]. Similarly mtDNA typing is used in mass disasters such as 9/11 [51].

## Gene loci used in taxonomy

The genetic loci of choice for forensic species identification are based on those derived from taxonomic and phylogenetic studies, and are primarily found on the mitochondrial genome [52]. Within mtDNA some gene sequences are thought to exhibit little intraspecific (within members of the same species) variability, but show sufficient interspecific (between different species) variation to allow for an estimation of degrees of relatedness and divergence times via calibrated molecular clocks. The main locus used in taxonomic and phylogenetic studies until recently was cytochrome *b *(cyt *b*) [53-55] which occurs between bases 14,747 and 15,887 in human mtDNA [56] and encodes a protein 380 amino acids in length. The cyt *b *locus has been used extensively in taxonomic and forensic studies [57-60], including tiger body parts [61-63], turtle eggs and shells [64-66], crocodile skins [67], rhino horn [68], elephant ivory [6], peafowl [69] and bear bile [11,63].

More recently, the use of cytochrome c oxidase I (COI) has increased owing primarily to its adoption by the Barcode for Life Consortium http://www.boldsystems.org[70,71]. COI is found between bases 5904 and 7445 in human mtDNA [56]. COI was used initially in the identification of invertebrate species [72-77]. It soon became the locus of choice in forensic entomology to identify the beetle larvae on a corpse [78,79]. As this one locus could identify these species, it was used more widely with the aim of being the locus of choice for identification of all animal species, to the extent that now the COI locus as a Barcode has been proposed for many types of organisms [80-87].

Other gene loci on the mitochondrial genome have been used in species identification. These include the 12S [88,89] and 16S rRNA loci [90] and the NDH family of genes [91-93]. The D-loop (displacement loop) has been used less in species identification but more in intraspecies identification [94,95]. Due to the greater sequence variation at this non-coding locus, it is now being used as a tool for identifying the presence of particular species within mixture of many species [96,97].

## Mitochondrial gene loci in species identification

**Figure 1:**
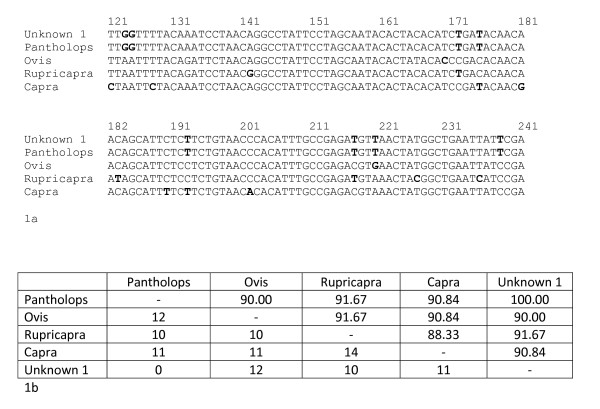
**Figure 1a and b showing a small part of the cyt b gene DNA sequence and their differences for four species** Figure 1a shows bases 121 – 241 of the cyt b gene for the Tibetan antelope (Pantholops hodgsonii: accession number AF034724) compared to the mammalian species with the closest homologies to this part of the sequence; being sheep (Ovis aries: accession AB0068000), the Pyrenean Chamoix  (Rupicapra pyrenaica: accession number AF034726) and a goat (Capra sumatrensis: accession number AY669321).  The unknown (or questioned) sequence comes from a shawl suspected as being from Shatoosh and derived from the Tibetan Antelope.  Figure 1b shows the number of bases differing between the four species (bottom of the rectangle) and the % similarity over the 120 bases. It would be normal to use over 400 bases in a similarity search rather than only the 120 as shown above but this indicates the process used in species testing.

The process of species identification in forensic science is becoming routine but has not been standardized to one single locus. Regardless of the locus used, the process is similar whereby the unknown, or questioned, sample is analysed by amplifying a section of the gene, predominantly a section of the cyt *b *gene or the COI gene. This polymerase chain reaction (PCR) fragment is then sequenced directly and the DNA sequence is compared to those registered on an open DNA databank such as GenBank [59]. It is unlikely that there will be a reference sample from a known species for direct comparison; hence there is a reliance on DNA sequence data on the database. GenBank currently has over 108 million sequences (as of August 2009) and, therefore, there is a high chance that the unknown sample will match a DNA sequence from a reference sample deposited on the database. If this is the case, and there is a 100% homology, then there is confidence that the unknown sample is a member of the species to which it matches. Figure 1 shows an example of the match between a sample taken from a shatoosh shawl, woven from the fine under hairs of the Tibetan antelope (*P. hodgsonii*), to the DNA sequence held on the GenBank database.

The availability of open access DNA sequence database, such as GenBank, has undoubtedly facilitated much scientific research and, in this regard, forensic science and species identification has also benefited. Allied to these benefits there is a certain amount of risk in any investigation, whether forensic or scientific. It is known that there are misidentified sequences on databases such as GenBank. For example, in a study of fungi, it was found that as much as 20% of the registered sequences may be misidentified [98]. DNA sequences registered with GenBank do not require that the sample comes from a voucher specimen in the first instance [99] as some museum samples considered to be voucher samples have been found to be misidentified [99]. As museum samples are often used as voucher specimens for sequences, a misidentified specimen in a museum may be very well be used as a 'voucher' sample for obtaining sequence data resulting in an erroneous sequence on the database.

Online and public databases are growing at an almost exponential rate. As more sequences are added to the database and, more importantly, more sequences from the same species are added, it will become easier to identify and remove misidentified sequences. Examples of this phenomenon can be seen in domesticated species (dog, cat, cow, chicken) where there may be up to 100 sequences for the same species for certain gene loci. If any one sequence is highly variable to all the others, then this aberrant sequence will be low on the list of matches. This not only shows the benefit of having multiple sequence data for the same species but also illustrates the potential for a misidentification if there is only one sequence for a particular species at a particular locus. In such a case it would be expected that this sequence would show a high degree of similarity to the DNA sequence from the next closest taxonomic species and, at least, the possibility of contamination from human sequence can be discarded.

If the DNA sequence from the unknown sample shows a 100% match to the reference sequence for *P. hodgsonii *and an 84% match to the next closest species, there is confidence that the unknown sample is that of the Tibetan antelope. This confidence that the questioned sample came from the Tibetan antelope and not any other species assumes: (1) that all species are held on the database and there is not another species of the same DNA sequence yet to be analysed; (2) all members of the Tibetan antelope have the same DNA sequence as that registered on GenBank (there is no intraspecies variation); and (3) the sequence data for the next closest match (in Figure 1 this is the goat) is also representative for this species and no members of this species have, by chance, the same sequence as that of the questioned sample. These three assumptions affect the confidence of species identification in any subsequent report.

Until recently, there had been no study to quantify intraspecies variation within the loci used in species identification. Such a study would address the above assumptions and provide a value for the confidence associated with a 100% match. It would also address the problem associated with a 99% or less match, in order to determine if any sequence variation could be due to intraspecies variation.

In the case of cyt *b *and COI, it is generally the practice to amplify a section of the genes for sequence analysis. Further, many of the samples examined are from material that may be at trace level, have many inhibitors to the PCR process present and be highly degraded. In the case of cyt *b *the section of the gene used is, typically, the first 400 bases [54,100-102] and in the case of COI a fragment of approximately 600 bases is used and no less than 500 base in length [103].

The primer sets that amplify a section of the cyt *b *gene were initially used by Pääbo *et al. *[101] and adapted by Kocher *et al. *[104] and later by Hseih *et al. *[100]. These primer sets were originally designed based on the human mitochondrial sequence [56,101]. Only later were they aligned with many cyt *b *gene DNA sequences and it was determined that there was high homology for many species. The aim was to develop a primer pair that would work on most, if not all, species regardless of their taxonomic order. Validation studies were based predominantly on using the primer sets to amplify numerous samples, sequence the PCR product, and compare to either sequence data on GenBank or from in-house sequence data. The universal primer sets for COI were designed by Folmer *et al. *[105] before being adopted by the Consortium for Barcode of Life.

## Cyt b and COI intraspecific and interspecific variations

For some time there has been a debate as to which of the cyt *b *or COI loci is the best gene for forensic species identification [106]. Advocates for each gene claim that their gene is better at identification of species but, until recently, there have not been any studies that could apply statistical confidences to sequence comparisons.

There are suggestions as to the levels of interspecific and intraspecific variation that should be expected given for each gene [82,107]. These values are generally based on a Kimura 2-parameter model (K2P) where the estimation of the number of nucleotide substitutions per site takes into account the different rates of transitions and transversions [108].

When the K2P model is used, authors generally state that intraspecifc variation is in the range of < 2-3% (7.93% between and 0.43% within bird species for COI [82]; 5.7% between and 1.5% within *Stenella *species for cyt *b *[54]). When anomalies arise these are interpreted as hidden or cryptic species [82], although these cryptic species may be based on levels of intraspecific variation from as few as two individuals [109]. This particular example relates to a study on North America bird species.

**Figure 2:**
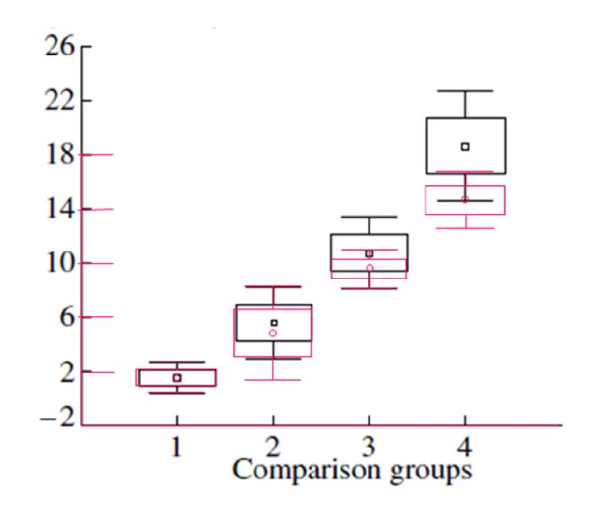
**An illustration of the variation on the *P *distances for both the cyt b (in black) and the COI (in red) gene loci using different taxonomic animal groups**. Group 1 is variation within a species; group 2 is variation between sibling species; group 3 is variation between species within the same genus; and group 4 is the variation between species of different genera but within the same Family. The central square indicates the mean, the larger box the standard error (SE) ± 1.00 and the bars either side of the boxes represent the SE ± 1.96. This graph has been adapted from that of Kartavtsev and Lee [108].

A study by Kartavtsev and Lee [110] investigated the nucleotide diversity between cyt *b *and COI at the population, genus and species levels. They analysed a wide range of vertebrate and invertebrate species but did not separate their results into different Classes and amalgamated all results. They determined that the closer any two samples were based on taxonomy, the closer they would be genetically, based on *p*-distance (similar to K2P but there is no distinction between the probabilities for transitions or transversions; Figure 2) [110].

The study by Kartavtsev and Lee [110] shows that suggested values for both genes appear to be correct. However, it is clear to see that there is overlap within COI of intraspecific variation and interspecific variation with sibling species (Figure 2).

A recent study by Tobe *et al*. [106,111], investigating only the Class Mammalia, refined these results further (for that Class) and derived a method to assign statistical confidence to sequence comparisons. In this study, the cyt *b *and COI gene sequences were compared for 217 different Mammalian species to assess interspecific variation and 945 human, 130 domestic cattle and 35 domestic dogs to assess intraspecific variation [111]. These data indicated a gap between the greatest observed intraspecific variation (1.5%) to the closest interspecific variation (2.5%), based on K2P values. A threshold can be applied given these data of a predicted maximum inter and intraspecific variation [111]. Both gene comparisons contained K2P values falling below 1.5% - 2.5% that, according to the sequence data, belonged to separate species, but these tended to be between sub-species or species with debated taxonomy [111].

By combining all data sets together close to 1 million K2P comparisons were made. As all sequence information was known, those comparisons between species and within species was known. From that data, Tobe *et al*. [111] were able to determine the rates of true positives, true negatives, false positives and false negatives at three different threshold values. Their results indicated that although both genes are similar in their discriminating power to separate species, cyt *b *performed better than did COI [111]. A K2P threshold of 1.5 showed that for COI the false positive rate was 4.85 × 10^-4 ^and the positive predictive value was 0.9995, whereas for cyt *b *the false positive rate was 2.02 × 10^-4 ^and the positive predictive value was 0.9998 [111]. These data indicate that both loci give a high degree of confidence in identification if the data falls within the intraspecific boundary but that there is an even lesser chance of a misidentification using cyt *b*.

As a further test of the two genes, Tobe *et al*. [111] constructed phylogenetic trees using the sequence data for each gene. They found that, no matter which tree building method was used, some species were always misplaced. Overall, the cyt *b *gene gave a more accurate reconstruction of Mammalian phylogeny at the Super Order, Order and Family levels than did COI [111].

Further testing for other Classes of organisms needs to be undertaken in order that any significance can be assigned to sequence comparisons. It is likely that the levels of intraspecific variation could be much greater in other, older, classes of organisms such as sharks or crocodiles.

## Reporting of results - current and future

In the absence of knowing about intraspecies and interspecies variation there is a limit to the confidence that can be reported as the outcome of a sequencing test to determine species. Currently, if there is a match with 100% homology between the questioned and reference sequence, there are two possibilities. Either the questioned sample is from the species that it matches or it matches by chance and comes from a species unknown that just happens to have the same DNA sequence as the questioned sample.

If there is not a 100% homology to any sequence on the database but there is a 99.5% homology to a sequence with two base differences between the two sequences over a total length of 400 bases and the next closest species has a 96% homology, then there are two most likely possibilities. Either (1) the questioned sample comes from the same species as that with a 99.5% homology and the differences are due to intraspecific variation or (2) it comes from an unknown but closely related species, with a 99.5% sequence match that has not been documented on the database.

However, many reporting this type of data would consider the possibility of such high intraspecies variation, such as exhibiting up to 96% similarity for a gene locus like cyt b or COI, is highly unlikely such that the only credible alternative is that the questioned sample is that from the species with the 99.5% homology. This is merely an assumption and can best be supported by studies on intraspecific variation.

With knowledge of intraspecific and interspecific variation detailed in the section above, the three scenarios above can be addressed with a probability. In all mammalian species examined there was a clear gap between intraspecies variation and interspecies variation. A false positive and false negative figure can be quoted allowing the confidence that a questioned sample is a member of the species to which it matches with a 99.5% homology.

The method of species testing is currently based on the sequence comparison of one mitochondrial locus: predominantly either the cyt *b *or COI locus. New methods of DNA sequencing open the prospect of sequencing whole genomes [112-114]. Any discussion over which of the two loci is more informative would no longer be valid as, instead of only 400 bases used in comparisons, 1000s of bases can be identified and compared. Validation studies are required in order to ensure that any sequence used in species identification still permits interspecies identification with a clear and unambiguous separation between one species and the next closest.

## Competing interests

The authors declare that they have no competing interests.

## Authors' contributions

The review was written with equal intellectual in-put by both authors.
